# Study on the differences of fat deposition in cattle-yak and yak based on transcriptomics and metabolomics

**DOI:** 10.3389/fvets.2025.1620146

**Published:** 2025-11-24

**Authors:** Lin Xiong, Jie Pei, Xingdong Wang, Shaoke Guo, Mengli Cao, Zhiqiang Ding, Yandong Kang, Xiaoyun Wu, Qianyun Ge, Xian Guo

**Affiliations:** 1 Key Laboratory of Yak Breeding in Gansu Province, Lanzhou Institute of Husbandry and Pharmaceutical Sciences, Chinese Academy of Agricultural Sciences, Lanzhou, China; 2 Key Laboratory of Animal Genetics and Breeding on Tibetan Plateau, Ministry of Agriculture and Rural Affairs, Lanzhou, China

**Keywords:** bovine, characteristics of fat deposition, regulatory gene, multi-omics, signaling pathway

## Abstract

The hybridization of yak with cattle is an effective means to improve the yak’s production performance. The fatty trait greatly affects the meat quality, the growth and development, and the reproduction of bovine. In this study, the thickness of subcutaneous fat in cattle-yaks and yaks was measured, and fatty acid composition was detected by a gas chromatograph-mass spectrometer (GC-MS); the transcriptome and metabolome in fat were detected by mRNA-Sequencing (mRNA-Seq) and an ultra-high performance liquid chromatography-mass spectrometry technique (UHPLC-MS/MS), respectively. The results revealed that the thickness of subcutaneous fat in yaks was greater than the value in cattle-yaks; the content of saturated fatty acids (SFAs), polyunsaturated fatty acids (PUFAs), and Σn-3 PUFAs in cattle-yaks’ fat were higher than the values in yaks’ fat, whereas the unsaturated fatty acids (UFAs) and monounsaturated fatty acids (MUFAs) content in cattle-yaks’ fat were lower. Furthermore, the expression of the *SREBF1* gene in the fat deposition of the two bovines was affected by PI3K-Akt and AMPK signal pathways, which led to the expression change of downstream *VLDLR*, *INSIG1*, *ACACA*, *LIPE*, *SLC2A4*, *CPT1C*, *SCD,* and *DGAT2* genes. Therefore, fatty acid synthesis, glucose and lipid transport, and lipid synthesis differed in fat depositions between the two bovines, ultimately leading to differences in the fat quantity in the two bovines. Moreover, the expression of *VLDLR*, *CPT1C*, *LEP*, *SCD,* and *CEBPE* genes was closely related to the differences in fatty acid composition between the fat tissues of two bovines. The results can provide some theoretical basis for yak breeding and can also promote the improvement of yak production.

## 1 Introduction

Fat is essential for glucose and insulin metabolism and hormone regulation (1), and plays an important role in the energy balance, temperature maintenance, endocrine, immune, and product quality of livestock (2, 3). Meanwhile, the intramuscular fat is closely related to the appearance, texture, flavor, juiciness, and hardness of bovine meat (4, 5) and plays an essential part in avoiding cold shortening, drip loss, and dark cutting. Fat deposition is the dynamic equilibrium controlled by many complex biological processes, such as adipocyte differentiation, the regulation of transcription factors and adipocytokines, triglyceride synthesis, and hydrolysis (6). Genetic factors (breed, sex, and genetic polymorphism), feeding management system (castration, feeding method, and dietary nutrition level), age, and hormone levels can affect the feature of fat deposition in bovines (7). The proliferation and differentiation of adipocytes are caused by the transformation of gene expression (8). The expression of lipogenic genes greatly affects the fat metabolism in bovine adipocytes, and there are significant differences in the regulation mechanism of fat deposition among different kinds of bovines (9).

Yak is the main breed of animal husbandry on the Qinghai-Tibet Plateau, and its meat, milk, and other products are important sources for the development of the local economy (10), but its production performance and economic benefits are lower than those of common cattle (11). Cattle-yak is the filial generation of yak (*Bos grunniens*) and cattle (*Bos taurus*) and shows superior heterosis to its parents (12, 13). The cattle-yak’s average daily gain, body length and height, carcass weight, and breast girth are higher than the yak’s values (14, 15), and cattle-yak also possesses stronger rough feeding and disease resistance (16). Moreover, the cattle-yak’s meat is characterized by high protein and low fat and is richer in the essential amino acids for human physiology, polyunsaturated fatty acids (PUFAs), and special flavor substances. Therefore, the hybrid F1 cattle-yak is an effective way to improve the production benefits of yak breeding and possesses broad market prospects and economic benefits (17, 18). In recent times, with the development of the cattle industry and the increase in human demand for high-quality meat, traditional yak breeding is being combined with modern cattle breeding, and the economic hybridization of yak with cattle is being accepted by more and more herdsmen and farms. Moreover, the research trends of cow and beef cattle are currently aimed at using the correlation techniques to improve milk, beef quality, and production at present. The studies on the cattle-yak’s and yak’s fat are in accord with the above global trends and possess a significant meaning to yak’s production.

The reports on cattle-yak are still very scarce at present, especially since the regulatory mechanism of fat deposition in cattle-yak is unknown. The composition of fatty acids is one of the characteristics of bovines. The transcriptomics based on high-throughput sequencing has been widely used to study the fat deposition in cattle (19), buffalo (20), and yak (21). Metabolomics was used to fill the gap between genes and phenotypes, and the many achievements in the study of bovines’ fat were obtained by this method (22). Metabolomics can quantitatively analyze these endogenous metabolites in bovines and has been successfully used to explore the fat traits in cows (23, 24). The transcriptomics based on mRNA-Seq can study the transcription status and transcriptional regulation rules of genes at an overall level and explore the key regulatory genes for the fat deposition of yak (25), cow (26), and beef cattle (27). However, it is often impossible to fully reveal the internal mechanism of fat deposition in bovines by a single omics dataset. Analyzing the economic traits of livestock by integrating omics techniques is more comprehensive and reliable, and this strategy greatly improves the progress of livestock breeding (28, 29). The key genes and signaling pathways regulating fat deposition in bovines can be revealed by the conjoint analysis of the transcriptome and metabolome in adipose tissue.

In this study, the thickness of subcutaneous fat in the waist and back of yaks and cattle-yaks was measured, and the fatty acid composition in subcutaneous fat was detected by gas chromatograph-mass spectrometer (GC–MS). Then, the different features of the fat deposition in cattle-yaks and yaks were explored. Furthermore, the transcriptome and metabolome in subcutaneous fat were detected by mRNA-Sequence (mRNA-Seq) and ultra-high-performance liquid chromatography-mass spectrometry technique (UHPLC–MS/MS), respectively. Finally, quantitative reverse transcriptase-polymerase chain reaction (qPCR) was performed to validate the differential expression of these selected genes identified by mRNA-Seq. The differentially expressed genes (DEGs) and different metabolites (DMs) were screened, and the biological functions of DEGs and DMs were analyzed by gene ontology (GO) and Kyoto encyclopedia of genes and genomes (KEGG) enrichment. Finally, the crucial genes and signaling pathways resulting in the differences in fat deposition between the two bovines were excavated through the association analysis of metabolome, transcriptome data, and fat phenotypic data. This study can establish a new theoretical basis for comprehensively revealing the regulatory mechanism of fat deposition in bovines as well as promote the breeding of yak’s new variety and the development of yak’s industrialization.

## 2 Materials and methods

### 2.1 Animals and samples collection

Six cattle-yaks (male, 4 years old, born to Jersey cattle-cross-Gannan yak) and six yaks (male, 4 years old, Gannan yak) were chosen as experimental animals. The feed experiment was carried out in the natural pasture in Xiahe County in Gansu Province, China. There is no specific breed of yak for meat at present. In the process of production practice, male yak is primarily used to produce meat, whereas female yak is primarily used to produce milk and reproduction. The cattle-yak born to Jersey cattle-cross-yak accounts for the relatively large proportion of cattle-yak production in China at present. Especially, the cattle-yak in Gansu province is mainly from the hybridization of female yak with male Jersey by artificial insemination. The female cattle-yak born to Jersey cattle-cross-yak is for dairy type, and the male cattle-yak born to Jersey cattle-cross-yak is for meat type. Therefore, the research method, in which male cattle-yaks born to Jersey cattle-yak crosses were chosen as the experimental animal, is representative and practical. All experimental bovines were kept in grazing conditions and could freely eat grass and drink water, and then, they were sacrificed through electrical stunning in late August. The subcutaneous fat samples on the surface of *longissimus dorsi* (12th–13th rib level) from each yak and cattle-yak were collected and then were divided into two parts. One was kept into liquid nitrogen for transcriptome and metabolome analysis, and the other was kept in a fridge at −20 °C for fatty acids analysis.

### 2.2 Measurement of subcutaneous fat thickness in bovine

The thickness of the subcutaneous fat on the back (the midline on both sides of the dorsal at the 5–6 *thoracic vertebrae*) and on the waist (both sides of the midline at the cruciate region) of cattle-yaks and yaks was measured using a Vernier caliper (Hengliang Inc., Shanghai) within 10 min after slaughter, respectively.

### 2.3 Determination of mRNA transcriptome in subcutaneous fat of bovine

The total RNA in the bovine’s subcutaneous fat was extracted using the mirVana^TM^ miRNA Isolation Kit (Ambion Inc., Foster City, CA, United States) following the manufacturer’s protocol (30). The extracted RNA’s integrity was analyzed using the Tanon 2500 agarose gel electrophoresis imager (31), and the RNA’s purity was detected using the ultraviolet spectrophotometer (Nanodrop 2000, Thermo) (32). The mRNA library for sequencing was prepared using the TruSeq Stranded mRNA LT Sample Prep Kit (Illumina, San Diego, CA, United States) (33). The library was sequenced on the Illumina sequencing platform (HiSeq^TM^ 2500), and 125 bp paired-end reads were generated.

### 2.4 Determination of metabolome in subcutaneous fat of bovine

#### 2.4.1 Metabolites extraction

A total of 30 mg of fat sample, two small steel balls, and 400 μL of the solution of methanol and methanol–water (4:1, *v:v*) containing mixed internal standard (4 μg/mL) were put into a 1.5-mL Eppendorf (EP) tube in sequence. The tube was precooled for 2 min and the sample was ground in a Wonbio-E grinder. The mixture was extracted with an F-060SD ultrasonic cleaner and then was left standing overnight. After being centrifuged at 12,000 r/min for 10 min, 150 mL supernatant was transferred and filtered through a 0.22-μm microfilter.

#### 2.4.2 Mass spectrum (MS) data collection

The Waters ACQUITY UPLC I-Class plus/Thermo QE plus with ACQUITY UPLC HSS T3 (100 mm × 2.1 mm, 1.8 μm) was used to collect the metabolite data. The elution solution consisted of A, the water containing 0.1% formic acid (*v*:*v*), and B, acetonitrile. The elution program was as follows: 5% B over 0.0–2.0 min, 5–30% B over 2.0–4.0 min, 30–50% B over 4.0–8.0 min, 50–80% B over 8.0–10.0 min, 80–100% B over 10.0–14.0 min, holding at 100% over 14.0–15.0 min, 100 to 5% B over 15.0–15.1 min, and holding at 5% B from 15.1 to 16.0 min. The flow rate, column temperature, and injection volume were 0.35 mL/min, 45 °C, and 3 μL, respectively. The MS system was operated using the ESI+ and ESI− mode, and the parameters were as follows: spray voltage 3,800 V (ESI+) and −3,000 V (ESI−), capillary temperature of 320 °C, aux gas heater temperature of 350 °C, a sheath gas flow rate of 35 Arb, an aux gas flow rate of 8 Arb, S-lens RF level 50, mass range 70–1,050 m/z, full ms resolution 70,000, MS/MS resolution 17,500, and normalized collision energy/stepped normalized collision energy 10, 20, 40.

### 2.5 Determination of fatty acids in subcutaneous fat of bovine

#### 2.5.1 Adipolysis and fatty acids derivatization

A total of 1 g of fat was taken into the tube with plugs. Methanol and potassium hydroxide were added to the tube, and then, the mixture was shaken for 2 h. The solution pH was adjusted to 3 using hydrochloric acid, after which 10 mL *n*-hexane was added to the solution and the tube was left standing for 10 min after shaking. The supernatant was dried under nitrogen. A total of 2 mL 1% sulfuric acid-methanol solution was added to the tube. The mixture was hydrolyzed for 30 min at 80 °C water bath and then fatty acid methyl esters (FAMEs) were extracted with 2 mL *n*-hexane. Two mL saturated salt solution was added, followed by shaking and centrifugation at 3,500 r/min for 2 min; then, the supernatant was transferred into the other tube. Twenty-five μL methyl nonadecanoate was added as the internal standard; then, the mixture was dried under nitrogen. The residue was redissolved in 1 mL n-hexane and the solution was filtered into a vial.

#### 2.5.2 Determination of fatty acid methyl esters (FAMEs)

GC–MS can separate the complex mixtures of fatty acids at high resolution and sensitivity and has been widely used to detect the fatty acids’ composition in cows (34) and beef cattle (35). An Agilent 7890/5975 GC–MS coupled with Agilent DB-WAX capillary-column chromatography (30 m × 0.25 mm ID × 0.25 μm) was used to analyze the extracts. GC parameters were as follows: the initial temperature of the column oven was 50 °C for 3 min, increased to 220 °C at 10 °C/min and held for 5 min, injection volume of 1 μL, split/splitless injector, and carrier gas helium at 1.0 mL/min. MS parameters were as follows: inlet temperature of 280 °C, ion source temperature of 230 °C, transmission line temperature of 250 °C, electron bombardment ionization (EI) source, SIM scanning mode, and electron energy 70 eV. The quality control sample was set to detect and evaluate the stability and repeatability of the system. The content of fatty acids was calculated by the external standard method using the mixed standard solution including 20 FAMEs.

### 2.6 Determination of gene expression by quantitative reverse transcription PCR (qPCR)

Each reverse transcription (RT) reaction in 10 μL consisted of 0.5 μg RNA, 2 μL 5 × TransScript All-in-one SuperMix for qPCR, and 0.5 μL gDNA Remover. Reactions were performed in the GeneAmp® PCR System 9,700 (Applied Biosystems, United States) for 15 min at 42 °C, 5 s at 85 °C. Then, the RT reaction mixture was diluted ×10 in nuclease-free water and held at −20 °C. Real-time PCR was performed using the LightCycler® 480 II Real-time PCR instrument (Roche, Switzerland) with a 10-μL PCR reaction mixture, including 1 μL cDNA, 5 μL 2 × PerfectStart^TM^ Green qPCR SuperMix, 0.2 μL forward primer, 0.2 μL reverse primer, and 3.6 μL nuclease-free water. Reaction was incubated in a 384-well optical plate (Roche, Swiss) at 94 °C for 30 s, followed by 45 cycles of 94 °C for 5 s and 60 °C for 30 s. The relative level of gene expression was calculated by the 2^-∆∆Ct^ method.

### 2.7 Statistical analyses

Fat thickness was analyzed with the independent-sample *t*-test in SPSS 16.0, and a *p*-value of <0.05 was considered to be a significant difference. Transcriptome data were pretreated with Trimmomatic; then, the clean reads were mapped to the yak’s reference genome. Fragments per kilobase of exon model per million mapped fragments (FPKMs) of genes were calculated with Cufflinks, and the gene read count was obtained with htseq-count. DEGs were identified using the DESeq (2012) R package functions by estimating SizeFactors and nbinomTest, and a *p*-value of < 0.05 and a foldchange (FC) of > 2 or < 0.5 were set as the threshold for DEGs. The GO and KEGG analyses for DEGs enrichment were also performed with R. Metabolome data were preprocessed with Progenesis QI v2.3. The combined date of positive and negative ions was analyzed with the R ropls package. The metabolites with variable importance in the projection (VIP) > 1.0 and *p* < 0.05 were selected as the DMs. Principal component analysis (PCA), orthogonal partial least squares discriminant analysis (OPLS-DA), and KEGG enrichment analysis were performed using R-based tools. The correlations between crucial DMs’ abundance, fatty acids’ content, and the expression level of crucial DEGs were evaluated by Pearson correlation analysis, respectively. The *p*-value of <0.05 and correlation coefficient >0.8 were the threshold values for the significant difference and high correlation.

## 3 Results

### 3.1 Differentially expressed genes (DEGs) and gene ontology (GO) and Kyoto encyclopedia of genes and genomes (KEGG) enrichment

**Figure 1:**
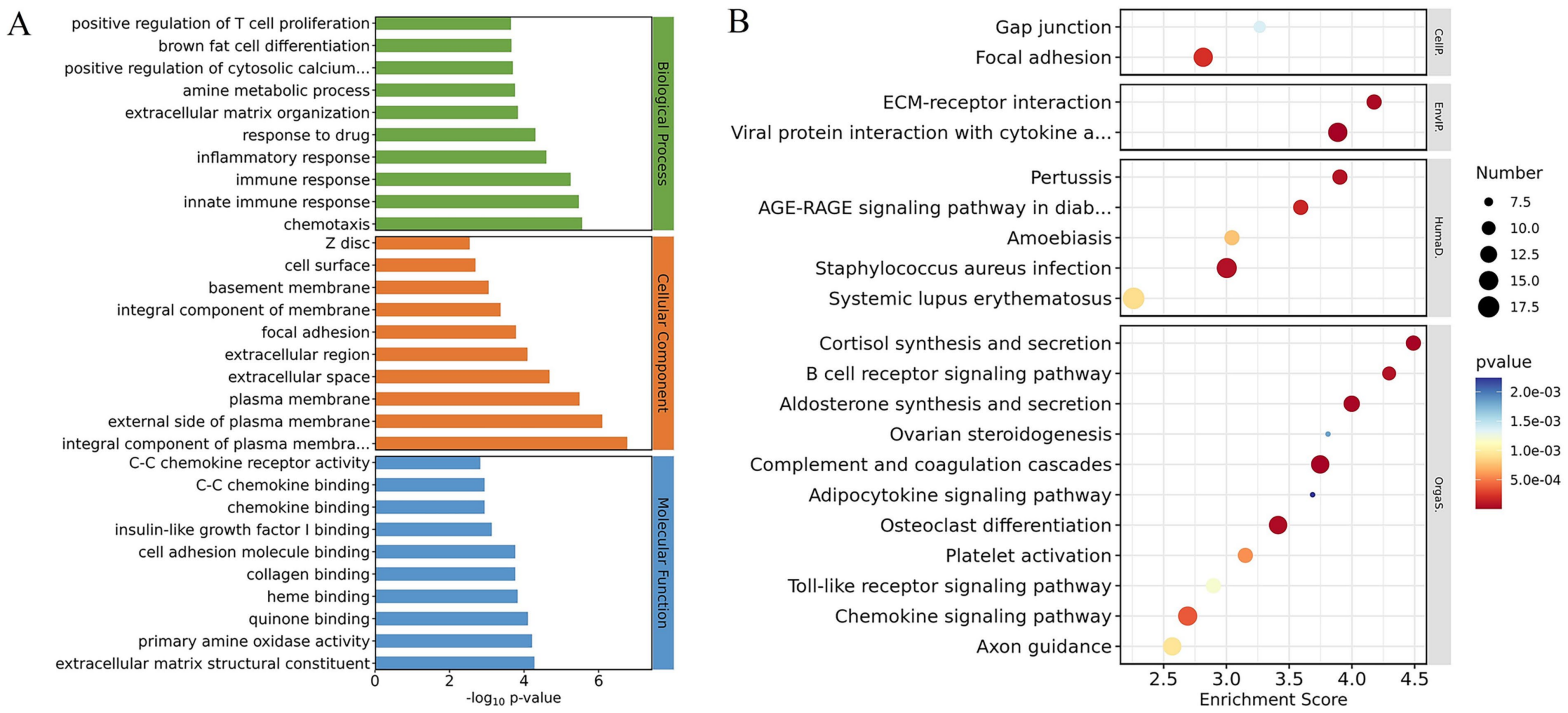
The differentially expressed genes (DEGs) in cattle-yak’s and yak’s fat and gene ontology (GO) term and Kyoto encyclopedia of genes and genomes (KEGG) pathways for DEGs enrichment. **(A)** The histogram of GO terms for DEGs enrichment. Horizontal and vertical axes represented the names of GO terms and the value of −log_10_ *P*, respectively. The green, red, and blue terms were related to biological process, cellular component, and molecular function, respectively. **(B)** The bubble diagram of the top 20 KEGG pathways for DEGs enrichment. The horizontal axis represented the enrichment score. The larger the item bubble was, the more DEGs the item contained. With the change of bubble color (purple-blue-green-red), the enrichment value gradually dwindled, and the differences were more significant.

A total of 1,216 DEGs were screened out in the cattle-yaks’ and yaks’ fat (Supplementary Table 1). The expression level of 679 genes was upregulated in the cattle-yaks’ fat, whereas the expression level of 537 genes was downregulated. The crucial information of DEGs on fat metabolism in the two bovines is shown in Table 1, and the diagram of the interaction network for these genes is shown in Supplementary Figure 1. GO enrichment showed that the DEGs were mainly involved in extracellular matrix organization, positive regulation of cytosolic calcium ion concentration, brown fat cell differentiation, cellular response to fatty acid, negative regulation of glucose import, cholesterol metabolic process, and adipose tissue development (Figure 1A; Supplementary Table 2). Furthermore, KEGG enrichment showed that the DEGs were mainly enriched in cortisol synthesis and secretion, aldosterone synthesis and secretion, extracellular matrix (ECM)-receptor interaction, focal adhesion, adipocytokine signaling pathway, PI3K-Akt signaling pathway, and AMPK signaling pathway (Figure 1B; Supplementary Table 3).

**Table 1:** The information on differentially expressed genes (DEGs) on fat metabolism in the cattle-yaks’ and yaks’ subcutaneous fat.

Gene ID	FC	*p*	Gene symbol
ENSBGRG00000004545	0.48	3.16E-04	*VLDLR*
ENSBGRG00000004885	2.02	1.77E-03	*ACADL*
ENSBGRG00000005349	0.25	3.36E-03	*FASN*
ENSBGRG00000005861	0.27	1.00E-04	*LDLR*
ENSBGRG00000005883	0.42	1.38E-03	*LPL*
ENSBGRG00000008633	0.12	2.12E-09	*ACSS2*
ENSBGRG00000010766	0.09	7.27E-04	*INSIG1*
ENSBGRG00000014792	0.22	1.07E-05	*ACACA*
ENSBGRG00000015027	0.20	1.12E-08	*ME1*
ENSBGRG00000018545	3.00	5.48E-06	*LIPE*
ENSBGRG00000025897	0.36	9.29E-03	*AGPAT2*
ENSBGRG00000000724	0.16	2.26E-08	*PRKAG3*
ENSBGRG00000023249	0.10	2.36E-37	*LEP*
ENSBGRG00000021445	0.34	1.99E-03	*SLC2A4*
ENSBGRG00000026150	0.41	9.45E-09	*MAST3*
ENSBGRG00000013548	0.44	3.22E-03	*SREBF1*
ENSBGRG00000023169	0.14	1.52E-10	*CPT1C*
ENSBGRG00000015740	3.02	1.73E-05	*DNMT3A*
ENSBGRG00000024230	0.14	1.54E-06	*SCD*
ENSBGRG00000026070	2.27	0.05	*PIK3AP1*
ENSBGRG00000017136	0.32	7.44E-06	*DGAT2*
ENSBGRG00000006600	22.18	0.00052	*CEBPE*
ENSBGRG00000004885	2.02	0.002	*ACADL*
ENSBGRG00000002007	0.17	3.03E-08	*ELOVL6*
ENSBGRG00000012620	3.53	9.98E-12	*ACAA1*

FC, Foldchange.

### 3.2 Different metabolites (DMs) and KEGG enrichment

**Figure 2:**
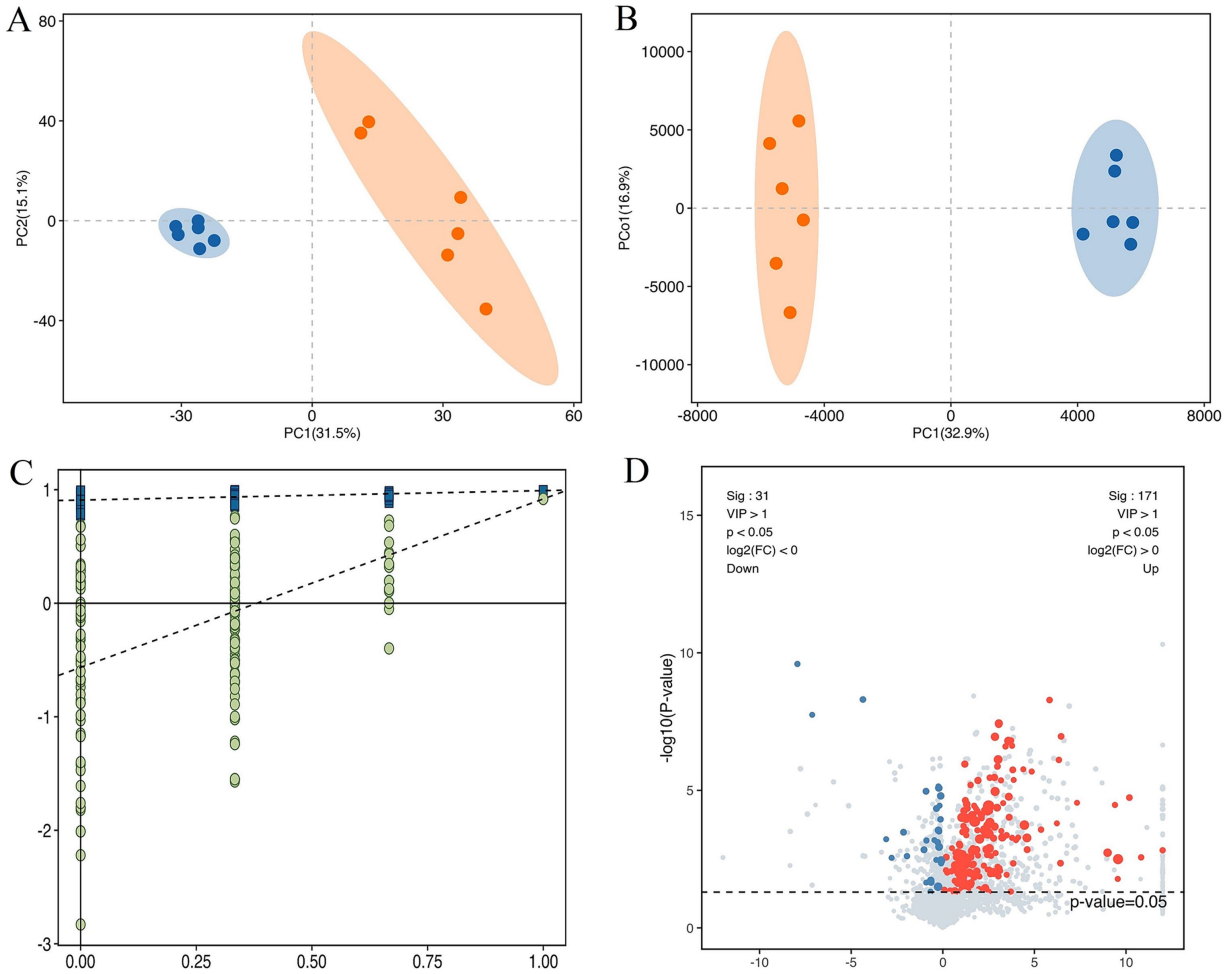
The different metabolites (DMs) in the cattle-yak’s and yak’s fat. **(A)** The score plot of principal component analysis (PCA) for the metabolites in the cattle-yaks’ and yaks’ subcutaneous fat. Blue and red circles represented the yak’s, cattle-yak’s fat, respectively. **(B)** The score plot of orthogonal partial least squares discriminant analysis (OPLS-DA) for metabolite. **(C)** The permutation test for OPLS-DA model. **(D)** The volcano plot of DMs in cattle-yak’s fat by contrast with yak’s fat. Abscissa represented the value of log_2_FC, and blue and red dots represented the downregulated and upregulated DMs in cattle-yak’s fat, respectively.

**Figure 3:**
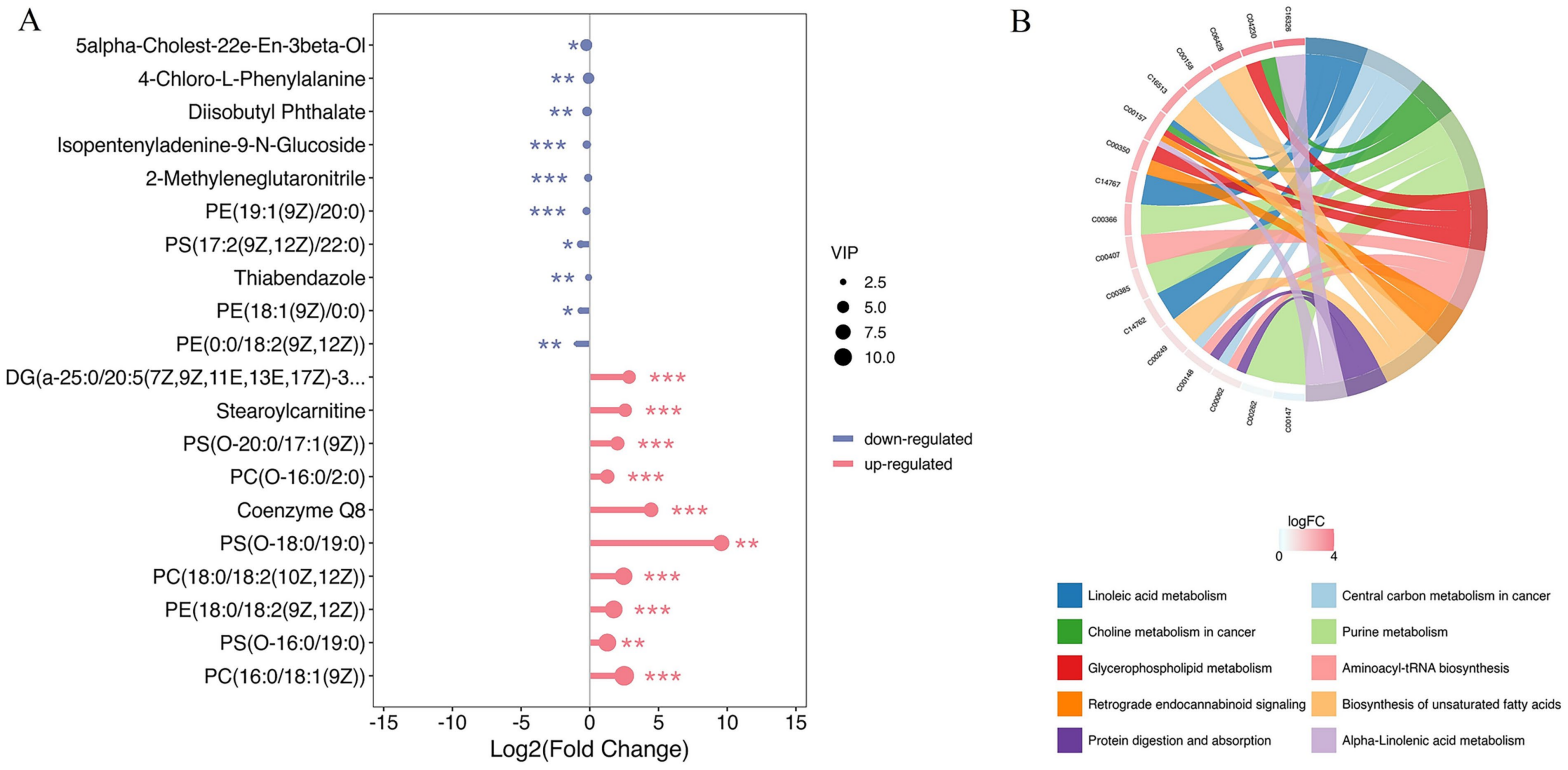
The lolipopmap for DMs and the chord diagrams of KEGG pathways. **(A)** The lolipopmap for DMs. The horizontal and vertical coordinates represented log_2_FC and DMs, respectively. The 10 DMs with the largest VIP value in the upregulated or downregulated DMs were drawn, respectively. The red and blue represented the upregulated and downregulated DMs in the cattle-yak’s fat, respectively. * indicated 0.01 < *p* < 0.05; ** indicated 0.001 < *p* < 0.01; *** indicated *p* < 0.001. Dot size was determined by VIP value. **(B)** The chord diagrams of KEGG pathways for DMs enrichment. The horizontal coordinates represented the enrichment score, and the vertical coordinates represented the information of the top 20 pathways. The larger the bubble was, the more DMs the KEGG pathway contained. With the bubble color changing from blue to red, the enrichment *p*-value gradually dwindled.

The score plots of PCA and OPLS-DA for the metabolites in cattle-yaks’ and yaks’ fat are shown in Figures 2A,B, respectively. It was found that the samples in two groups were clearly differentiated, which indicated that there were obvious differences in the metabolites between cattle-yaks’ and yaks’ fat. The testing using 200 random permutations was used to validate the OPLS-DA models (Figure 2C). The value of R^2^Y and vertical intercept was 0.909 and −0.565, respectively, which showed that the model possessed better stability and that there was no overfitting phenomenon. Therefore, the model was effective and stable, and the hybridization indeed induced the marked perturbation of metabolites in the yak’s subcutaneous fat. The volcano plot of metabolites in cattle-yaks’ fat, by contrast with yaks’ fat, is shown in Figure 2D. A total of 202 DMs were screened out (Supplementary Table 4), and the abundance of 171 DMs was upregulated in cattle-yaks’ fat, whereas the abundance of 31 DMs was downregulated. Lolipopmap is similar to a bar chart, but its expression is more intuitive and the chart form is richer, so it is used to more intuitively display the DMs and log_2_ FC values (Figure 3A). The KEGG pathways for DMs enrichment were mainly related to linoleic acid metabolism, glycerophospholipid metabolism, PPAR signaling pathway, biosynthesis of unsaturated fatty acids, and mTOR signaling pathway (Supplementary Table 5). The important DMs on the fat deposition of two kinds of bovines are shown in Table 2. The top 10 KEGG pathways with listHits value > 1 and the lowest *p*-value were selected to draw the chord diagrams (Figure 3B).

**Table 2:** The information on important different metabolites (DMs) in cattle-yaks’ and yaks’ fat.

Metabolite	Class	VIP	FC
PC(16:0/18:1(9Z))	Glycerophospholipids	12.48	5.67
13 s-Hode	Fatty acyls	1.98	2.12
9 s-Hode	Fatty acyls	1.78	5.38
PE(18:0/18:1(11Z))	Glycerophospholipids	4.62	5.43
PC(18:3(6Z,9Z,12Z)/0:0)	Glycerophospholipids	1.72	13.20
Palmitic acid	Fatty acyls	3.24	1.99
EPA	Fatty acyls	4.78	11.97
DPA	Fatty acyls	1.88	7.92
L-Arginine	Carboxylic acids and derivatives	1.72	1.63
L-Proline	Carboxylic acids and derivatives	1.77	1.82
2 s-Amino-3 s-methylpentanoic acid	Carboxylic acids and derivatives	5.46	3.06
Sphingosine	Organonitrogen compounds	1.43	2.84

VIP, variable important in the projection.

### 3.3 Thickness and fatty acids composition in cattle-yaks’ and yaks’ fat

The subcutaneous fat thickness in cattle-yaks’ back, 3.93 ± 0.22 mm, was less than the value, 4.41 ± 0.54 mm, in yaks’ back (*p* < 0.05), and the subcutaneous fat thickness in cattle-yaks’ waist, 4.39 ± 0.39 mm, was less than the value, 5.01 ± 0.75 mm, in yaks’ waist too. A total of 17 fatty acids were simultaneously detected in the cattle-yaks’ and yaks’ fat (Table 3), which included five five SFAs, five MUFAs, and seven PUFAs. Nine fatty acids’ content was different in cattle-yaks’ and yaks’ fat, which included *cis*-C18:3n3, *cis*-C20:5n3, *trans*-C18:1, *cis*-C22:1, C17:0, C15:0, C18:0, *cis*-C18:1, and *cis*-C16:1. Of them, C18:3n3, *cis*-C20:5n3, *trans*-C18:1, *cis*-C22:1, C17:0, C15:0, C18:0, and *cis*-C18:1 contents in cattle-yaks’ fat were higher than the value in yaks’ fat. Moreover, ΣSFAs, ΣPUFAs, and Σn-3PUFAs contents and ΣPUFAs/ΣSFAs ratio in cattle-yaks’ fat were higher than the values in yaks’ fat, whereas ΣUFAs and ΣMUFAs contents and Σn-6/Σn-3 PUFAs ratio in cattle-yaks’ fat were lower.

**Table 3:** Comparison of fatty acid composition in cattle-yaks’ and yaks’ fat.

Fatty acid	Cattle-yak (mean ± SD, %)	Yak (mean ± SD, %)
*cis*-C17:1	8.14 ± 0.74	7.56 ± 1.33
*cis*-C20:2n6 (LA)	0.23 ± 0.04	0.17 ± 0.07
*cis*-C18:3n3 (ALA)	5.27 ± 0.66 ^A^	0.70 ± 0.30 ^B^
*cis*-C18:3n6 (GLA)	0.02 ± 0.01	0.01 ± 0.0004
*cis*-C20:5n3 (EPA)	0.72 ± 0.20 ^A^	0.08 ± 0.02 ^B^
*trans*-C18:1	10.56 ± 1.40 ^A^	4.56 ± 1.66 ^B^
C20:4n6 (ARA)	2.83 ± 0.97	2.63 ± 0.49
*cis*-C22:1	0.05 ± 0.01 ^A^	0.03 ± 0.01 ^B^
C17:0	1.26 ± 0.17 ^A^	0.74 ± 0.25 ^B^
C13:0	ND	ND
C15:0	0.59 ± 0.11 ^A^	0.30 ± 0.10 ^B^
C11:0	ND	ND
*cis*-C18:2n6 (LA)	0.45 ± 0.17	0.48 ± 0.14
C18:0	14.64 ± 2.18 ^A^	9.35 ± 2.79 ^B^
*cis*-C18:1	0.95 ± 0.12 ^A^	0.41 ± 0.15 ^B^
*trans*-Traumatic acid	0.002 ± 0.008	0.001 ± 0.007
C12:0	0.34 ± 0.30	0.15 ± 0.04
C10:0	ND	ND
C16:0	20.16 ± 2.09	18.83 ± 2.21
*cis*-C16:1	33.74 ± 5.04 ^A^	54.01 ± 8.62 ^B^
ΣSFAs	37.01 ± 4.55 ^a^	29.36 ± 5.08 ^b^
ΣUFAs	62.99 ± 4.55 ^a^	70.64 ± 5.08 ^b^
ΣPUFAs	9.52 ± 1.94 ^A^	4.08 ± 0.90 ^B^
ΣMUFAs	53.47 ± 4.57 ^A^	66.56 ± 5.90 ^B^
Σn-6 PUFAs	3.53 ± 1.15	3.30 ± 0.67
Σn-3 PUFAs	5.99 ± 0.84 ^A^	0.78 ± 0.27 ^B^
Σn-6/Σn-3 PUFAs	0.58 ± 0.14 ^A^	4.70 ± 1.72 ^B^
ΣPUFAs/ΣSFAs	0.26 ± 0.07 ^A^	0.14 ± 0.04 ^B^

ΣMUFAs, sum of monounsaturated fatty acids; ΣSFAs, sum of saturated fatty acids; ΣPUFAs, sum of polyunsaturated fatty acids; ΣUFAs, sum of unsaturated fatty acids. ND, no detection. The values in the same row with different lowercase superscripts showed a *p*-value of <0.05, and the values in the same row with different capital superscripts showed a *p*-value of <0.01.

### 3.4 Results of correlation analysis

**Figure 4:**
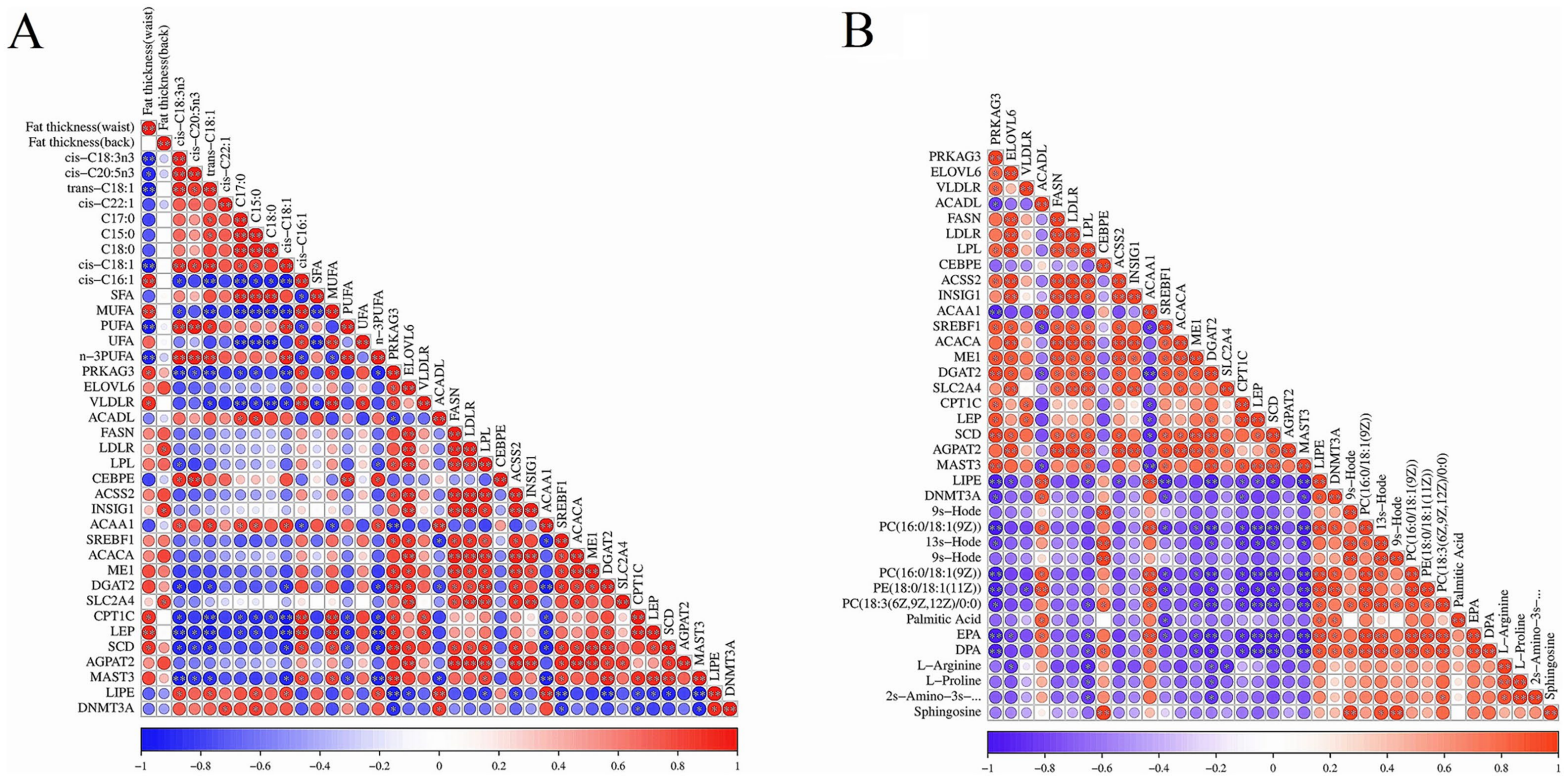
The results of correlation analysis between DEGs and fat, DMs in two bovines’ fat. **(A)** The heat map of Pearson correlation between important DEGs and different fatty acids and fat thickness. Red and blue indicated positive and negative correlation between these DEGs and fatty acid and fat thickness, respectively. The color depth represented the correlation coefficient. The darker the color was, the higher the correlation; the dot size represented correlation significance. **(B)** The heat map of Pearson correlation between important DEGs and DMs in yak’s and cattle-yak’s fat.

The heat map for the Pearson correlation between the important DEGs’ expression abundance and the different fatty acids and fat thickness is shown in Figure 4A. There were highly positive correlations among most of the DEGs, except *ACAA1*, *ACADL,* and *LIPE* genes. The fat thickness was highly positively correlated with the expression level of *PRKAG3*, *VLDLR*, *LDLR*, *INSIG1*, *SLC2A4*, *CPT1*, *LEP,* and *SCD* genes; ΣSFAs content was negatively correlated with the expression of the *VLDLR* gene; ΣMUFAs was highly positively correlated with the expression of *PRKAG3*, *VLDLR*, *CPT1*, *LEP,* and *SCD* genes; ΣPUFAs content was highly positively correlated with the expression of the *CEBPE* gene; whereas, it was negatively correlated with the expression of *CPT1*, *LEP*, *SCD,* and *MAST3* genes. Moreover, the expression of *PRKAG3*, *VLDLR*, *CPT1*, *LEP*, *SCD,* and *MAST3* genes was negatively correlated with the most different fatty acids, whereas the expression of *ACAA1* and *LIPE* genes was negatively correlated with the most different fatty acids. The heat map of Pearson correlation for DEGs with DMs is shown in Figure 4B. The expression of *ACADL*, *CEBPE*, *ACAA1*, *LIPE,* and *DNMT3A* genes was positively correlated with the most crucial DMs abundance, whereas the expression of other DEGs was negatively correlated with the most crucial DMs abundance.

### 3.5 DEGs validation by qPCR

To confirm the reproducibility of the gene expression abundance from mRNA-Seq, eight DEGs (*SCD*, *CPT1*, *FASN*, *SREBF1*, *LIPE*, *ACAA1*, *DGAT2,* and *AGPAT2*) on fat metabolism were randomly chosen for qPCR verification. The Yak *β-actin* gene was selected as the reference gene. The primer information for the eight DEGs is shown in Supplementary Table 6. The qPCR analysis showed that the expression level of *SCD*, *CPT1*, *FASN*, *SREBF1*, *DGAT2,* and *AGPAT2* genes was downregulated in cattle-yaks’ fat, whereas the expression of *LIPE* and *ACAA1* genes was upregulated. Therefore, the expression trends of DEGs from mRNA-Seq and qPCR were similar, and the reliability of the sequencing data was confirmed.

## 4 Discussion

**Figure 5:**
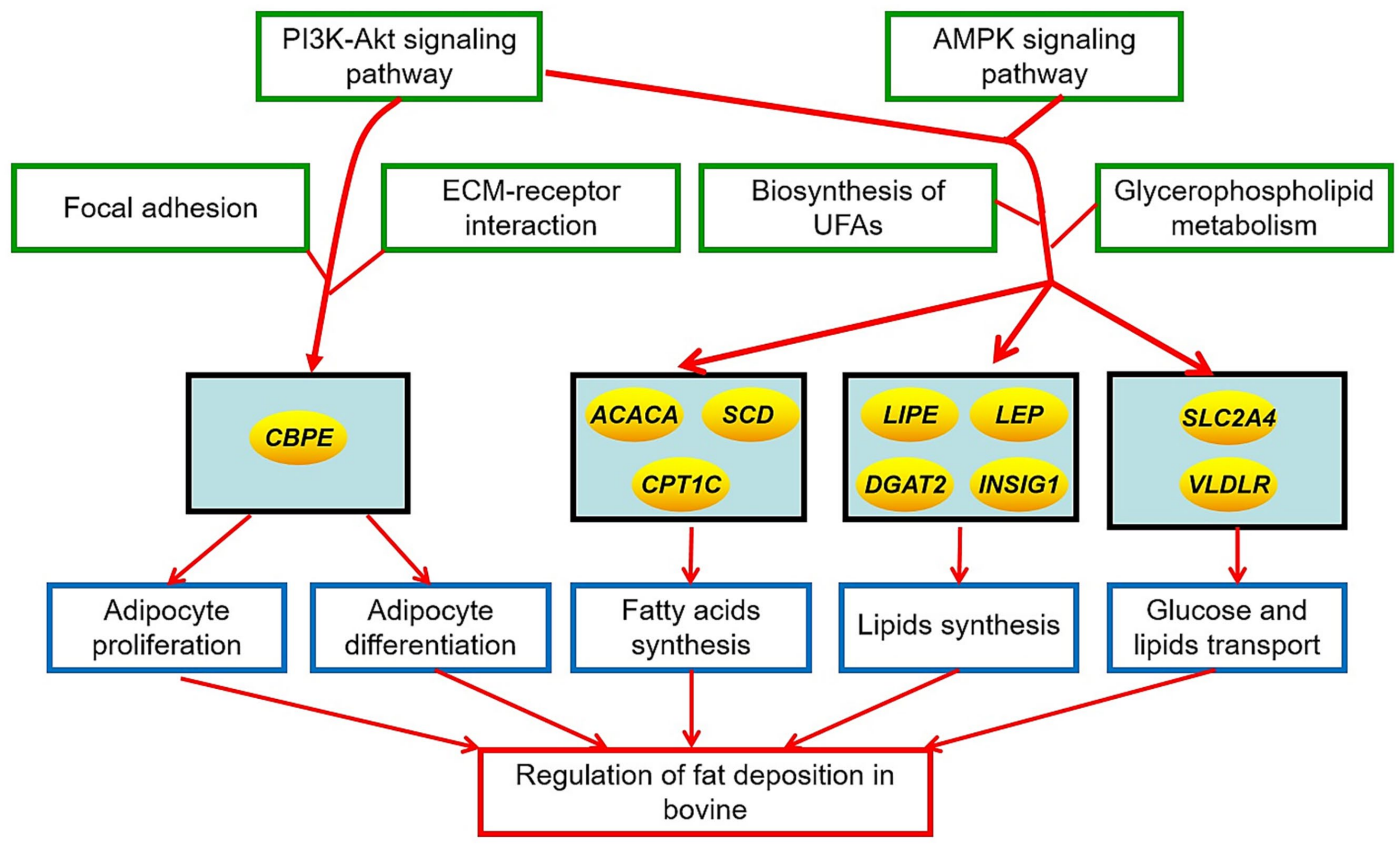
The action mechanism of different fat deposition in yak and cattle-yak. The ellipse represented the key genes regulating fat deposition in two bovines. Green boxes represented the crucial signal pathways regulating fat deposition in two bovines. Blue boxes represented the crucial metabolic process affecting the fat deposition in two bovines.

The total fat quantity and fatty acid composition in cattle-yak and yak were significantly different. The DEGs on fat metabolism primarily included *PRKAG3*, *VLDLR*, *FASN*, *LDLR*, *INSIG1*, *ACACA*, *LIPE*, *SLC2A4*, *CPT1C*, *DGAT2*, *CPT-1*, *LEP*, *SCD,* and *CEBPE*, and the KEGG pathways for the DEGs enrichment mainly involved adipocyte differentiation and proliferation, PI3K-AKT, and AMPK signaling pathway. These DMs on fat metabolism are mainly glycerophospholipids and fatty acyls, and the KEGG pathways for DM enrichment were mainly involved in UFAs and glycerophospholipids metabolism. Furthermore, there was a high correlation in the expression level or content between the many DEGs regulating fat metabolism and fat quantity, UFAs, SFAs, PUFAs, MUFAs, and the most DMs in the fat tissues of the two bovines. The results showed that the differences in fat features in two bovines were closely related to these DMs and were regulated by the above DEGs. The action mechanism for the differences in fat deposition in yak and cattle-yak is shown in Figure 5.

The subcutaneous fat’s thickness in yak was greater than the value in cattle-yak, so it was preliminarily inferred that the deposition capacity of the subcutaneous fat in yak was stronger than that of cattle-yak born to Jersey cattle-cross-Gannan yak. The DEGs were mainly related to hormone secretion, adipocyte growth, and energy regulation, and the DMs were mainly related to lipid metabolism and adipocyte growth regulation. A total of 19 DMs, being glycerophospholipids, were enriched in glycerophospholipid metabolism pathways, mainly including PC (16:0/18:1(9Z)), PE (18:0/18:1(11Z)), and some of these DMs can be transferred into glycerol-3-phosphate. Triacylglycerol biosynthesis is derived from glycerol-3-phosphate as a precursor (36). Linoleic acid metabolism is one of the mechanisms for fatty acid synthesis and decomposition and can regulate blood sugar level, activate the glycolipid transporters on the cell membrane to transport fatty acids into the cell, promote the oxidation of SFAs, and reduce the cholesterol and triacylglycerol content (37). Glycerol phospholipid metabolism plays an important role in fat deposition too (38). Therefore, it can be inferred that the differences in fat deposition in cattle-yak and yak were closely related to the glycerophospholipid metabolism and self-difference of adipocyte.

The PI3K-AKT and AMPK signaling pathways can activate the expression of upstream or downstream genes by exogenous signals like the environment, growth factors, and nutrient levels. The intake nutrients can be used to synthesize lipids, and these lipids are transported in the form of low-density lipoprotein. Then, the process of insulin secretion is stimulated, which activates the PI3K-Akt pathway. The expression of these genes on lipid synthesis is affected (39), and the *de novo* synthesis of fatty acids, promotion of adipocyte differentiation, inhibition of lipolysis, and reduction of free fatty acids in adipose tissue are realized. In addition, ECM is an important part of the focal adhesions pathway and PI3K-AKT signaling pathway and plays an important role in fat deposition. Focal adhesions are the cell-matrix adhesion structures mediated by integrins and promote the strong attachment to the matrix (40). The adipocytokine signaling pathway is a significant contributor to the development of muscle marbling in cattle (41). Moreover, the AMPK signaling pathway is also implicated in glucose and fatty acid metabolism (42). The regulation of lipid metabolism by the AMPK signaling pathway includes the decrease in lipogenesis and the stimulation of mitochondrial fatty acid oxidation. There are also complex interactions between PI3K-Akt and AMPK signal pathways (43, 44). AMPK possesses two-way regulation of the PI3K-Akt signal pathway, and AMPK activation promotes RS, PI3K, and Akt activation. Therefore, the differences in fat deposition in cattle-yak and yak were closely related to PI3K-AKT and AMPK signaling pathways.

The PI3K-Akt signal pathway can affect lipid synthesis, transport, storage, and degradation by acting on SREBPs (45). SREBPs regulate the expression of *SCD1*, *FASN*, *ACC,* and *HMG-CoA* genes, which control lipid, cholesterol ester, and triglyceride synthesis (46). SREBPs also promote the conversion of citric acid to acetyl-CoA by regulating the expression level of *ACLY* and *ACSS2* genes; then, the *de novo* synthesis of fatty acids is strengthened. Moreover, the PI3K-Akt signal pathway can also raise the expression of the *LDLR* gene by acting on SREBPs and then promote cholesterol intake and affects lipid transport. AMPK can promote glucose uptake and lipid oxidation by increasing the expression of *ACC* and *CPT-1* genes (47) and inhibits lipid synthesis by decreasing the expression of *SREBP-1*, *FASN*, and *SCD* genes. In mammals, the activated AMPK can inhibit SREBP (48), which decreases the expression level of lipogenic genes, including *FASN*, *ACC1,* and *SCD,* impacting UFAs biosynthesis (49). The overexpression of the *SCD1* gene can lead to excessive fat deposition; the expression of the *CPT1A* gene promotes fatty acid oxidation and lipid synthesis; the *FASN* gene is closely related to fatty acid synthesis and fat deposition; and the overexpression of the *DGAT2* gene can stimulate lipid droplet formation and triacylglycerol accumulation in bovines (50). PI3K-Akt and AMPK signal pathways also regulate glucose transport by GLUT4. The decreased expression of the *SLC2A4* gene in cattle fat could result in decreased efficiency glucose metabolism (51). The PI3K-Akt signaling pathway is essential for cell proliferation and apoptosis, and the *CEBPE* gene is a candidate gene for differentiation in cattle adipocytes (52). Moreover, the fat thickness in yak and cattle-yak was highly positively correlated with the expression of *PRKAG3*, *VLDLR*, *LDLR*, *INSIG1*, *SLC2A4*, *CPT1*, *LEP,* and *SCD* genes. Leptin is the upstream binding factor of AMPK, and the upregulated expression of the *LEP* gene can activate the AMPK signaling pathway. INSIG1 is associated with fat metabolism and adipocyte differentiation in Buffalo (53) and can regulate SREBP activation. Moreover, *INSIG1* is the target gene of SREBPs, and its expression is regulated by SREBPs. The *VLDLR* gene plays an important role in regulating body weight and fat-related traits, as well. PRKAG3 encodes the gamma-3 subunit of AMPK and negatively regulates the intramuscular fat deposition in livestock (54). Therefore, the difference in fat quantity between yak and cattle-yak was closely related to the expression of *PRKAG3*, *VLDLR*, *LDLR*, *INSIG1*, *SLC2A4*, *CPT1*, *LEP,* and *SCD* genes.

The fatty acid composition in beef cattle is influenced by genetic factors. The ΣSFAs content in cattle-yak’s fat was higher than the value in yak’s fat, whereas the ΣUFAs content in cattle-yak’s fat was lower. Meanwhile, ΣSFAs content in fat of the two bovines was negatively correlated with the expression level of the *VLDLR* gene. It was reported that the expression level of the *VLDLR* gene was negatively associated with the concentration of SFAs in cattle serum (55). A total of three DMs, including palmitic acid, EPA, and DPA, were enriched in UFAs biosynthesis, so it was inferred that these fatty acids’ metabolism greatly affects the composition of UFAs in cattle-yak’s and yak’s fat. Fatter bovine possesses a higher percentage of ΣMUFAs in meat, whereas the percentage of ΣPUFAs is lower (56, 57), which is consistent with the feature of fat deposition in yak and cattle-yak too. The longest-chain MUFAs are derived from palmitic acids by the elongation or oxidation of the carbon chain. The MUFAs content in two bovines’ fat was highly positively correlated with the expression level of *PRKAG3*, *VLDLR*, *CPT1*, *LEP,* and *SCD* genes. The *SCD* gene is a key gene regulating MUFAs synthesis, and C16:0 and C18:0 can be converted into C16:1 and C18:1 under the dehydrogenation of the *SCD* gene (58). The PUFAs in livestock cannot be directly synthesized *in vivo* and must be derived from the precursor compounds in feed or grass. The n-6 PUFAs are derived from linoleic acid, while the n-3 PUFAs are derived from linolenic acid. The PUFAs content in two bovines’ fat was highly positively correlated with the expression level of the *CEBPE* gene, whereas it was negatively correlated with the expression level of *CPT1*, *LEP*, *SCD,* and *MAST3* genes. CCAAT/enhancer binding protein is a key transcription factor regulating the terminal differentiation of adipocytes (59), and the *CEBPE* gene plays an important role in the differences in PUFAs in fat deposition between two bovines. *LEP* and *SCD* genes are considered to be the candidate genes regulating the PUFAs content in sheep muscle (60). The *cis*-C18:3n3, *cis*-C20:5n3, ΣPUFAs, and Σn-3PUFAs contents in cattle-yak’s fat were higher, and 13 kinds of glycerophospholipids and two kinds of fatty acyls were enriched in linoleic acid metabolism. EPA and DPA can be derived from ALA and further transferred into DHA and prostaglandin (61). Therefore, it was inferred that the differences in fatty acid composition between two bovines’ fat were closely related to the expression level of *VLDLR*, *CPT-1*, *LEP*, *SCD,* and *CEBPE* genes.

## 5 Conclusion

The capacity of fat deposition in yak was stronger than that of cattle-yak born to Jersey cattle-cross-yak. Meanwhile, the composition of ΣSFAs, ΣMUFAs, ΣPUFAs, and Σn-3 PUFAs in cattle-yak’s and yak’s fat was significantly different. The glycerophospholipid metabolism and adipocyte growth under the action of PI3K-Akt and AMPK signal pathway resulted in the differences in fat deposition between two bovines. The *SREBF1* gene played an important role of mediation in this process. *VLDLR*, *FASN*, *LDLR*, *INSIG1*, *ACACA*, *LIPE*, *SLC2A4*, *CPT1C*, *SCD,* and *DGAT2* genes may be considered to be the important candidates for regulating the fat quantity in bovines, and the fatty acid composition in bovine’s fat may be regulated by *VLDLR*, *CPT-1*, *LEP*, *SCD,* and *CEBPE* genes. The above genes possess excellent potential in breeding new varieties of yak in the future, and yaks with stronger fat deposition and a higher percentage of functional fatty acids may be obtained based on the above crucial genes.

## Data Availability

The datasets generated for this study can be found in the Sequence Read Archive (https://www.ncbi.nlm.nih.gov/sra) at NCBI, with the BioProject ID: PRJNA1268695.

## Supplementary material

SUPPLEMENTARY FIGURE 1 The diagram of interaction network for the crucial DEGs on the two bovines’ fat metabolism.

SUPPLEMENTARY TABLE 1 The information of DEGs in yak-cattle’s and yak’s fat.

SUPPLEMENTARY TABLE 2 The information of GO term for DEGs enrichment.

SUPPLEMENTARY TABLE 3 The information of KEGG pathways for DEGs enrichment.

SUPPLEMENTARY TABLE 4 The information of DMs in yak-cattle’s and yak’s fat.

SUPPLEMENTARY TABLE 5 The information of KEGG pathways for DMs enrichment.

SUPPLEMENTARY TABLE 6 The primer information for SCD, CPT1, FASN, SREBF1, LIPE, ACAA1, DGAT2 and AGPAT2 genes in qPCR.
