# Supplementary material for: Study on the differences of fat deposition in cattle-yak and yak based on transcriptomics and metabolomics
Source: Front Vet Sci. 2025 Nov 24;12:1620146. doi: 10.3389/fvets.2025.1620146 (PMC12683920; doi:10.3389/fvets.2025.1620146)
Supplement: SUPPLEMENTARY FIGURE 1 — The diagram of interaction network for the crucial DEGs on the two bovines’ fat metabolism. [file Image_1.pdf]

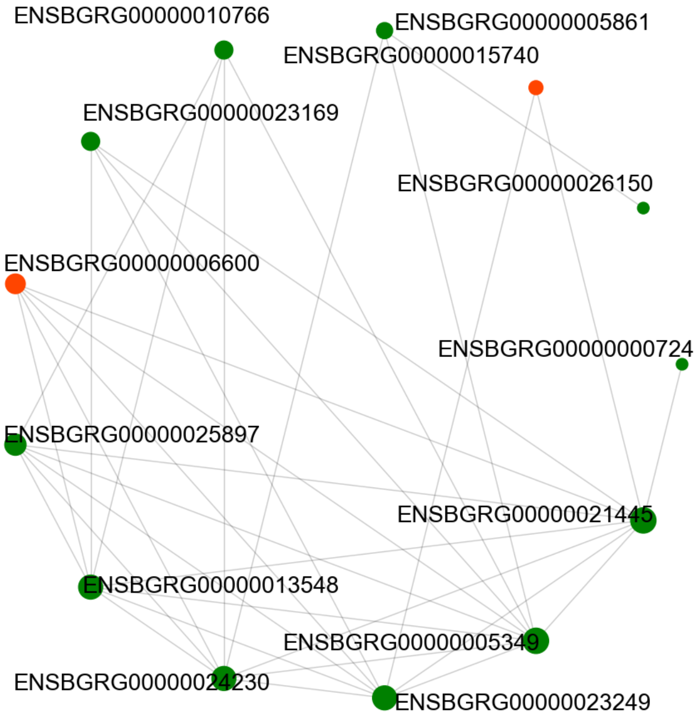

Supplementary Figure 1 The diagram of interaction network for the crucial DEGs on the two bovines' fat metabolism. The red represented the up-regulated DEGs in the cattle-yaks' fat, whereas the green represented the down-regulated DEGs in the cattle-yaks' fat.
