# Supplementary material for: Study on the differences of fat deposition in cattle-yak and yak based on transcriptomics and metabolomics
Source: Front Vet Sci. 2025 Nov 24;12:1620146. doi: 10.3389/fvets.2025.1620146 (PMC12683920; doi:10.3389/fvets.2025.1620146)
Supplement: SUPPLEMENTARY TABLE 6 — The primer information for SCD, CPT1, FASN, SREBF1, LIPE, ACAA1, DGAT2 and AGPAT2 genes in qPCR. [file Table_6.docx]

Supplementary Table 6 The information of primer for the 8 selected differentially expressed genes (DEGs) in the subcutaneous fat of yak and cattle-yak.

| **Gene Symbol** | **Forward primer**  **(5->3)** | **Reverse primer (5->3)** | **Product length (bp)** | Tm  (℃) |
| --- | --- | --- | --- | --- |
| *β-actin* | GGATGCAGAAAGAGATCACT | TCTGCTGGAAGGTGGACA | 187 | 60 |
| *SCD* | TACCACGTTCTTCATTGATTGC | TGTAGCTTTCCTCTCCAGTT | 113 | 60 |
| *CPT1* | GCCAAGACCTTGTCTGGA | GAGATGGCAGCGTTTGAT | 84 | 60 |
| *FASN* | CCTACACTCAGAGCTACCG | TGCATGAAGAAGCACATGG | 82 | 60 |
| *SREBF1* | GCATTTCACCGAACCGAG | CTGGTGCACTCCGATCTACA | 87 | 60 |
| *DGAT2* | GGTCCTGGAGGTGAACTGA | AGCACTTGAGAACTTGGTAGA | 89 | 60 |
| *AGPAT2* | GACATGATGGGCCTCATGGA | CTGCCGGTTGATGAAGAGGA | 126 | 60 |
| LIPE | CTTCTTCGAGGGTGATGAG | CGGGTGTGAACTGGAAAC | 107 | 60 |
| ACAA1 | GGCTAGAGACTGCCTGAT | AAGCCAGTGCAAAGGTAT | 98 | 60 |
